# The cytotoxicity evaluation of magnetic iron oxide nanoparticles on human aortic endothelial cells

**DOI:** 10.1186/1556-276X-8-215

**Published:** 2013-05-07

**Authors:** Gaoyuan Ge, Hengfang Wu, Fei Xiong, Yu Zhang, Zhirui Guo, Zhiping Bian, Jindan Xu, Chunrong Gu, Ning Gu, Xiangjian Chen, Di Yang

**Affiliations:** 1 Research Institute of Cardiovascular Disease, First Affiliated Hospital of Nanjing Medical University, 300 Guangzhou Road, Nanjing 210029, China; 2 Department of Cardiology, First Affiliated Hospital of Nanjing Medical University, 300 Guangzhou Road, Nanjing 210029, China; 3 State Key Laboratory of Molecule and Biomolecule Electronics, Jiangsu Provincial Laboratory for Biomaterials and Devices, Southeast University, Nanjing, 210009, China; 4 Second Affiliated Hospital of Nanjing Medical University, Nanjing, 210011, China

**Keywords:** Magnetic nanoparticles, Iron oxide, Endothelial cells, Cell viability, Angiogenesis

## Abstract

One major obstacle for successful application of nanoparticles in medicine is its potential nanotoxicity on the environment and human health. In this study, we evaluated the cytotoxicity effect of dimercaptosuccinic acid-coated iron oxide (DMSA-Fe_2_O_3_) using cultured human aortic endothelial cells (HAECs). Our results showed that DMSA-Fe_2_O_3_ in the culture medium could be absorbed into HAECs, and dispersed in the cytoplasm. The cytotoxicity effect of DMSA-Fe_2_O_3_ on HAECs was dose-dependent, and the concentrations no more than 0.02 mg/ml had little toxic effect which were revealed by tetrazolium dye assay. Meanwhile, the cell injury biomarker, lactate dehydrogenase, was not significantly higher than that from control cells (without DMSA-Fe_2_O_3_). However, the endocrine function for endothelin-1 and prostacyclin I-2, as well as the urea transporter function, was altered even without obvious evidence of cell injury in this context. We also showed by real-time PCR analysis that DMSA-Fe_2_O_3_ exposure resulted in differential effects on the expressions of pro- and anti-apoptosis genes of HAECs. Meanwhile, it was noted that DMSA-Fe_2_O_3_ exposure could activate the expression of genes related to oxidative stress and adhesion molecules, which suggested that inflammatory response might be evoked. Moreover, we demonstrated by *in vitro* endothelial tube formation that even a small amount of DMSA-Fe_2_O_3_ (0.01 and 0.02 mg/ml) could inhibit angiogenesis by the HAECs. Altogether, these results indicate that DMSA-Fe_2_O_3_ have some cytotoxicity that may cause side effects on normal endothelial cells.

## Background

The application of magnetic nanoparticles (MNPs) in diagnosis and effective treatment of diseases has become an area of increasing interest in the biomedical sciences [1-4]. Drug delivery is used to carry drugs region-specifically by attaching them to MNPs and releasing the drug *in vivo* to the target locale [5-9]. Via AC magnetic fields, the MNPs can mediate hyperthermia for *in situ* cancer-targeted therapy and be used for *in vitro* cancer cell-targeted detecting systems [10-14]. Similarly, cells of interest labeling with large amounts of MNPs can be located, tracked, and recovered by imaging techniques such as high-resolution magnetic resonance imaging [15-18].

MNPs of iron oxide (Fe_3_O_4_, γ-Fe_2_O_3_) may develop to be the modest and biocompatible one with the rapid progress in biological applications research [19,20]. Many investigations have studied the use of diverse organic coatings as a way of optimizing the delivery of MNPs to or into cell. Several studies have confirmed that a simple dimercaptosuccinic acid (DMSA) coating can enhance the rate of uptake by three orders of magnitude, presumptively by engendering the MNPs with an anionic charge, leading to nonspecific adsorption to the cell surface followed by endocytosis into the cell [21-23]. These methods can deliver huge amounts of MNPs into the cells, but a proven concern arises over the impacts that great intracellular concentrations of MNPs might have on normal cell behavior. A quantitative model cell system indicates that intracellular delivery of even restrained levels of iron oxide (Fe_2_O_3_) nanoparticles may affect cell function. To be more specific, the cytotoxicity investigations show that exposure to mounting concentrations of anionic MNPs, from 0.15 to 15 mM of iron, results in a dose-dependent decreasing viability and capacity of PC12 cells to spread neurites in return for nerve growth factor [24].

In addition to drug delivery, many biomedical applications of MNPs such as magnetic tracking and hyperthermia need a very great deal of MNPs to be injected into blood vessels, which are lined by endothelial cells (ECs), a single squamous epithelial cell layer and an anticoagulant barrier between the vessel wall and blood. EC is involved in the immune and inflammatory response, coagulation, growth regulation, production of extracellular matrix components, and is a modulator of blood flow and blood vessel tone. EC injury, activation, or dysfunction is a hallmark of many pathologic states including atherosclerosis, loss of semi-permeable membrane function, and thrombosis [25]. A wide variety of stimuli can induce programmed cell death (apoptosis) of endothelial cells through extrinsic (death receptor) and/or intrinsic (mitochondria) apoptotic pathway, which is ultimately executed by the intracellular proteases called caspases. There also exist caspase-independent pathways of apoptosis and anti-apoptotic proteins, which can protect cells from apoptosis. These pathways and proteins compose the complicated network of the cell apoptosis [26-29]. When injecting MNPs into blood vessels, ECs is the first tissue barrier encountered by the MNPs. The focus of this study is thus on the cytotoxicity evaluation of DMSA-coated Fe_2_O_3_ nanoparticles (DMSA-Fe_2_O_3_) on human aortic endothelial cell (HAEC), which is able to proliferate for many generations maintaining its endothelial characteristic and is widely used for *in vitro* study [30].

## Methods

### Materials

Dulbecco's modified Eagle’s medium (DMEM) and fetal bovine serum (FBS) were purchased from GIBCO Company (Grand Island, New York, USA). Endothelial cell growth supplement (ECGS) was supplied by M&C Gene Technology (Beijing, China). MEM non-essential amino acid solution (100×), l-glutamine, thiazolyl blue tetrazolium bromide, haematoxylin, penicillin, and streptomycin were obtained from Sigma-Aldrich (St Louis, MO, USA). Prostacyclin I-2 (PGI-2), endothelin-1 (ET-1), and nitric oxide (NO) assay kits were obtained from Nanjing Jiancheng Bioengineering Institute (Nanjing, China). Primers were synthesized by Sangon Biotechnology Co., Ltd. (Shanghai, China), and RNAiso Plus reagent, PrimeScript™ RT reagent Kit, and SYBR Premix Ex Taq™ were from TaKaRa Biotechnology Co., Ltd. (Dalian, China). Matrigel basement membrane matrix was from Becton Dickinson (Bedford, MA, USA).

### Preparation of DMSA-Fe_2_O_3_ nanoparticles

The DMSA-Fe_2_O_3_ was prepared by co-authors Dr. Fei Xiong, Dr. Yu Zhang, and Dr. Ning Gu. The characterization data, such as transmission electronic microscopy (TEM) images, crystal structure, surface charge, and magnetic measurements and Fourier transform infrared spectroscopy measurements were determined as the previous report in Dr. Gu's Lab [31]. In the present study, quasi-spherical DMSA-Fe_2_O_3_ with an average diameter of 10 nm, was diluted in deionized water to 1 mg/ml, and then further diluted in tested concentrations with cell culture medium before using.

### Cell culture

HAECs were used for experiments at passages 2 to 5. HAECs were cultured in DMEM supplemented with 1% ECGS, 20% FBS, 1% heparin sodium, 1% non-essential amino acid solution (100×), 1% l-glutamine, 100 U/ml penicillin, and 100 U/ml streptomycin. Cells were maintained at 37°C in a humidified incubator with 5% CO_2_.

### Location of DMSA-Fe_2_O_3_ in the HAEC

For TEM analysis, the HAECs incubated with 0.02 mg/ml DMSA-Fe_2_O_3_ for 24 h were washed with PBS and routinely fixed, dehydrated, and embedded [32]. Ultrathin sections (80 nm) were transferred to the 200 mesh copper grid, stained with 5% lead tetraacetate, air-dried, and then examined with a TEM (JEM-1010, JEOL, Akishima-shi, Japan) at 80 kV.

### Cell viability/cytotoxicity assay

The cytotoxicity of DMSA-Fe_2_O_3_ against HAECs was investigated by the tetrazolium dye (MTT) assay [33]. For the dose-dependent effect, the DMSA-Fe_2_O_3_, diluted with culture medium at graded concentrations from 0.001 to 0.2 mg/ml, was applied to the HAECs for 24 h. For the time-dependent effect, 0.05 mg/ml of DMSA-Fe_2_O_3_ was applied to the cells for 4, 24, 48, and 72 h, respectively. After washing with PBS, the cells were incubated with MTT solution at 37°C for 2 h, and the dyes were dissolved by dimethyl sulfoxide (DMSO) for 15 min. Absorbance was examined at 595 nm with the Ultra Microplate Reader ELX808IU, and cell viability was calculated as a percentage of control cells treated without DMSA-Fe_2_O_3_. Each experiment was repeated at least three times independently.

### Assessments of HAEC injury markers and endocrine factors

In this study, HAECs were co-cultured with 0.02 mg/ml of DMSA-Fe_2_O_3_ for 24 h. Then, the cell culture supernatant was centrifuged at 8000 × *g*, 4°C for 30 min to remove the rest of the nanoparticles and cell debris. ET-1, PGI-2, and NO concentrations in the supernatant were measured using ELISA kits according to the manufacturer's instructions, respectively. Lactate dehydrogenase (LDH) and urea were determined using an automatic biochemistry analyzer (Olympus AU5400, Olympus Corporation, Shinjuku-ku, Japan).

### Real-time PCR analysis of HAEC gene expression

Thirty-eight genes related to apoptosis cascade, endoplasmic reticulum (ER) stress, oxidative stress, adhesion molecules, and calcium-handling proteins were detected by real-time PCR. In this study, HAECs were incubated with 0.02 mg/ml of DMSA-Fe_2_O_3_ for 24 h. The total RNA (300 ng) extracted from HAECs was reverse-transcribed using the PrimeScript™ RT reagent Kit, and then the cDNA was amplified using the SYBR Premix Ex Taq™ according to the following cycle conditions: 30 s at 95°C for 1 cycle, 5 s at 95°C, and 30 s at 60°C for 40 cycles (AB 7900HT Fast Real-Time PCR system). All real-time PCR reactions were performed in triplicate. The housekeeping gene GAPDH was used as an internal control. The fold changes of target gene expression relative to those of the control group were analyzed by the 2^-ΔΔCT^ method [34], divided into different ranges and depicted as different colors.

### Effects of DMSA-Fe_2_O_3_ on HAEC tube formation

The tube formation assay is one of the most widely used assays to model the reorganization stage of angiogenesis *in vitro*. The assay measures the ability of endothelial cells, plated at subconfluent densities with the appropriate extracellular matrix support, to form capillary-like structures. In this study, the Matrigel basement membrane matrix was used as extracellular matrix support to observe whether angiogenesis of HAEC can be intervened by DMSA-Fe_2_O_3_ or not. For HAEC tube formation, 50 μl/well of the Matrigel basement membrane matrix was added to a 96-well plate and allowed to gel for 60 min at 37°C. Then, HAECs were seeded at a density of 1.5 × 10^4^ cells/well on the surface of the gel in the presence or absence of conditioned DMSA-Fe_2_O_3_ and incubated for 14 h at 37°C in a CO_2_ incubator. Meanwhile, the high urea solution (6M urea) was used as a positive control for inhibition of tube formation. The cultures on the gel were fixed for 10 min in 25% glutaraldehyde, washed, and stained with Mayer’s hematoxylin. Each well was inspected under a light microscope at ×100 magnification and captured more than three pictures from different fields. Image-Pro plus (IPP) 6.0 for Windows software (Media Cybernetics, Inc., Rockville, MD, USA) was used to measure the length of tube formation on each picture. The average data from the same well was calculated as its quantitative value.

### Statistical analysis

The data were represented as mean ± SD of no less than three independent experiments. Statistical analysis was performed using a student's *t* test. A value of *p* < 0.05 was considered statistically significant.

## Results and discussion

### Endocytosis of DMSA-Fe_2_O_3_ by HAECs

**Figure 1:**
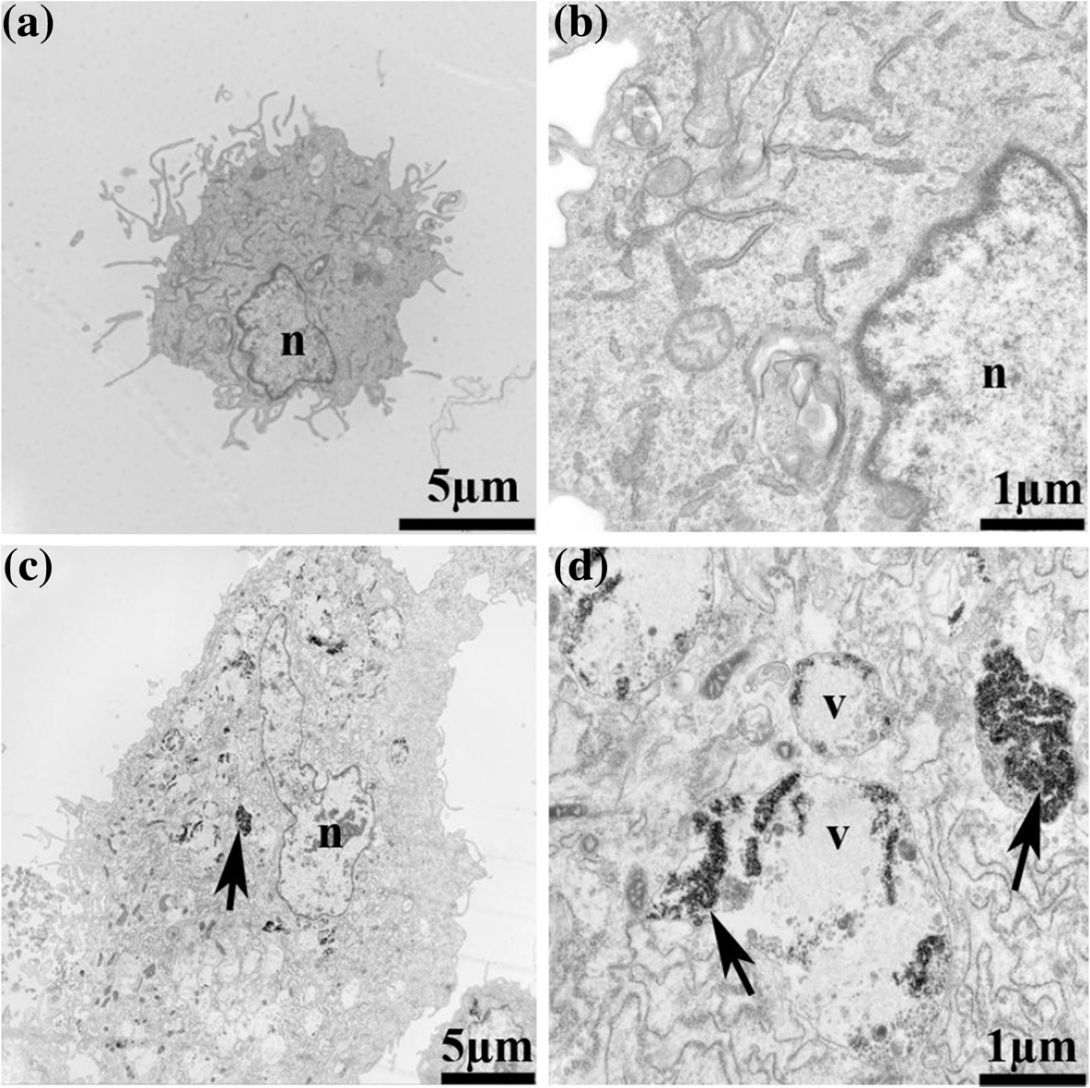
**The TEM images of HAECs incubated with 0.02 mg/ml of DMSA-Fe**_**2**_**O**_**3 **_**for 24 h. **(**a**) HAEC without DMSA-Fe_2_O_3 _(×8,000). (**b**) HAEC without DMSA-Fe_2_O_3 _(×30,000). (**c**) HAEC incubated with DMSA-Fe_2_O_3 _(×5,000). (**d**) HAEC incubated with DMSA-Fe_2_O_3 _(×30,000). Abbreviations: n, nucleus; v, vesicle; Arrows denote the DMSA-Fe_2_O_3 _or particulate matter.

We were able to recognize the DMSA-Fe_2_O_3_ inside the HAECs and distinguish them from the cellular structures by their high electron density on TEM. Figure 1 represents TEM micrographic images between HAECs incubation with 0.02 mg/ml of DMSA-Fe_2_O_3_ (Figure 1c,d) and HAECs without DMSA-Fe_2_O_3_ incubation (Figure 1a,b). The results indicate that the DMSA-Fe_2_O_3_ aggregates are readily absorbed by the cells without disrupting the integrity of the cellular membrane and dispersed in the cytoplasm.

### HAECs viability studies

The tetrazolium dye (MTT) assay has been used for detecting the number of viable cells (proliferation) and loss of viable cells (cytotoxicity) resulting from toxic materials since only living cells can reduce the MTT to its insoluble form, formazan, which can be quantitatively measured after dissolved in DMSO by a spectrophotometer, and the resultant value is linked to the number of living cells [35].

**Figure 2:**
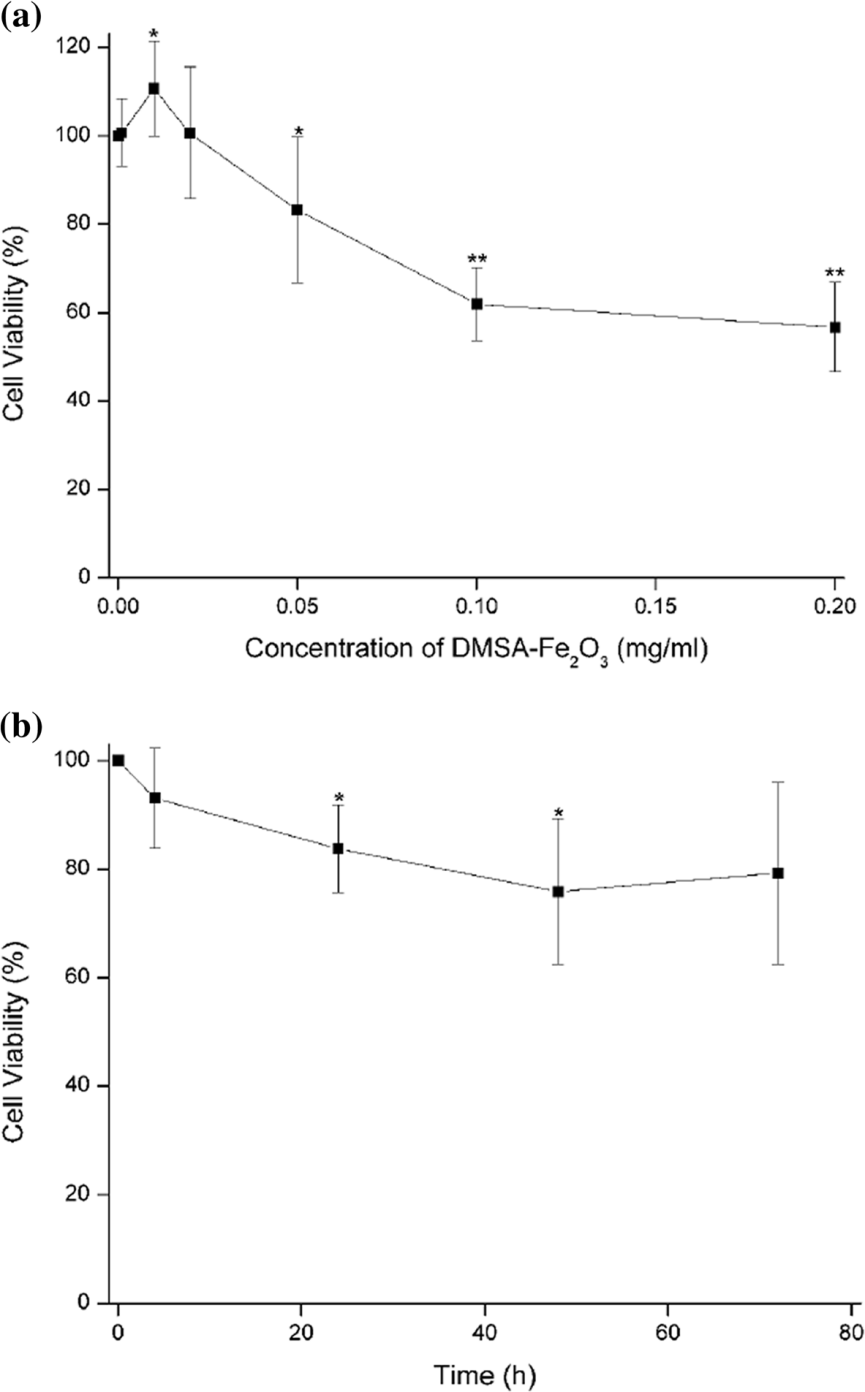
**The viability of HAECs incubated with DMSA-Fe**_**2**_**O**_**3**_**. **Data are expressed as mean ± SD from independent experiments. Control values from HAECs incubated without DMSA-Fe_2_O_3 _were defined as 1. (**a**) HAECs were incubated with DMEM containing the gradient concentrations of DMSA-Fe_2_O_3 _for 24 h (0.001, 0.01, 0.02, 0.05, 0.1, 0.2 mg/ml), *n *= 7. (**b**) HAECs were incubated with DMEM containing 0.05 mg/ml DMSA-Fe_2_O_3 _for the indicated time (4, 24, 48, 72 h). *n *= 5. *p < 0.05 vs. control; **p < 0.01 vs. control.

In the present study, the viability of HAECs was apparently decreased with increased DMSA-Fe_2_O_3_ concentrations compared with that of control cells (Figure 2a). HAECs treated with the concentrations under 0.05 mg/ml of DMSA-Fe_2_O_3_ for 24 h did not induce any cell losses. In contrast, DMSA-Fe_2_O_3_ at the high doses (greater than 0.05 mg/ml) resulted in significant cell loss thereby cytotoxic. The cell viability of HAECs incubated with DMSA-Fe_2_O_3_ at the concentration of 0.2 mg/ml was approximately decreased to 56.7% of the control cells.

To study the time-dependent effect of DMSA-Fe_2_O_3_ on HAECs viability, cells were incubated with 0.05 mg/ml of DMSA-Fe_2_O_3_ for 4, 24, 48, and 72 h, respectively (Figure 2b). Decreased cell viability occurred as early as 4 h and varied in a range from 75.8% to 93.1% to the control group at tested time points. The results suggest that the cytotoxic effect of DMSA-Fe_2_O_3_ on HAECs is dose-dependent, and the concentrations no more than 0.02 mg/ml are relatively harmless in the present study.

### Effects of DMSA-Fe_2_O_3_ on HAEC injury markers and endocrine factors

**Figure 3:**
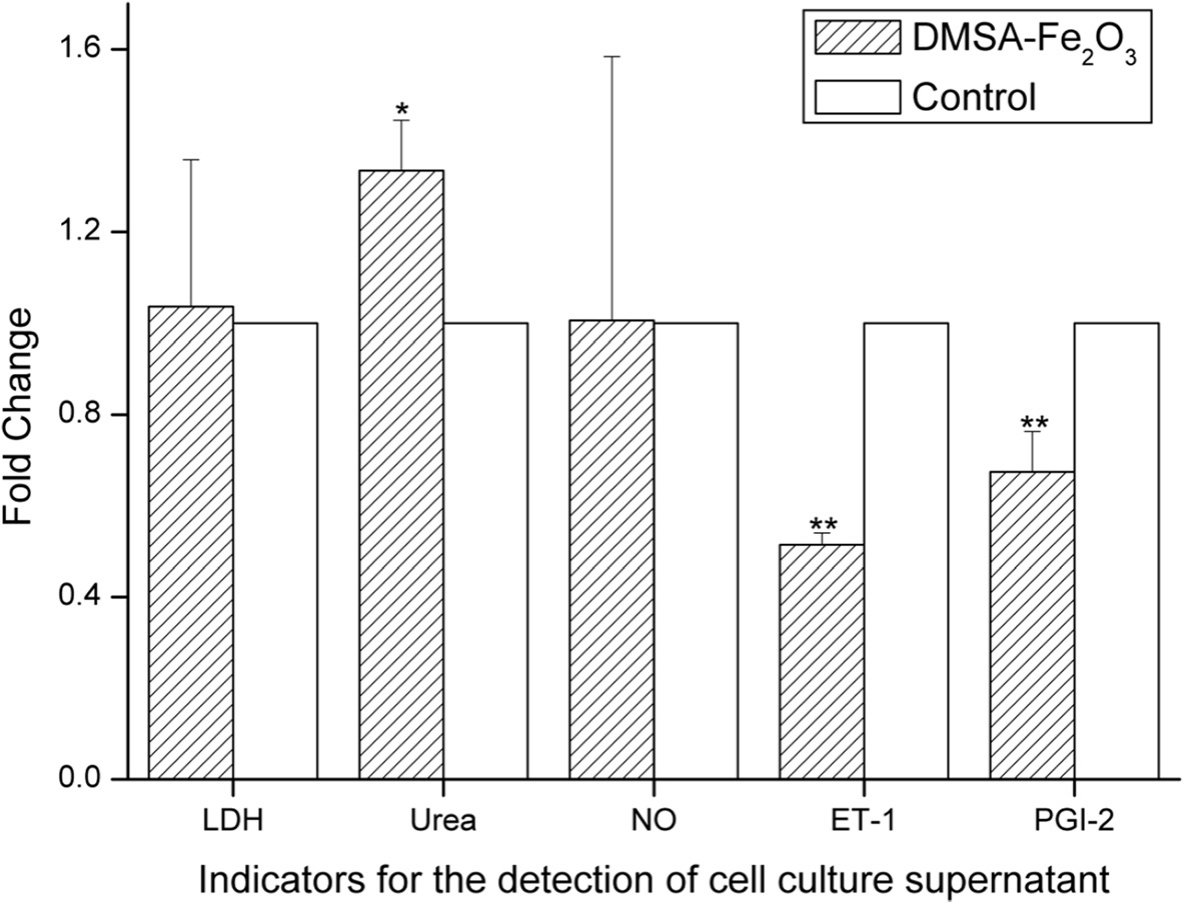
**Levels of injury marker, LDH, and endocrine factors in supernatant of HAECs.** Incubated with 0.02 mg/ml DMSA-Fe_2_O_3 _for 24 h. Ratios relative to the control cells (without DMSA-Fe_2_O_3_) are shown. *p < 0.05 vs. control; **p < 0.01 vs. control.

LDH is a cytoplasmic enzyme which can be released to the extracellular space because of the disturbances of the cellular integrity induced by pathological conditions. Therefore, supernatant LDH of cultured HAECs is detected as a marker for cell injury [36]. We found that there was no difference in LDH released from the HAECs incubated with 0.02 mg/ml DMSA-Fe_2_O_3_ for 24 h and the control cells (Figure 3). This finding was consistent with the results of little cytotoxicity effect in MTT assay (Figure 2a) and cell membrane integrity changes shown by TEM (Figure 1c,d).

We then examined whether the endocrine function of HAECs was changed when exposed to this low dose of DMSA-Fe_2_O_3_ that did not cause measurable cell injury. ECs can regulate blood pressure and blood flow by releasing vasodilators such as NO and PGI-2, as well as vasoconstrictors, including ET-1. So, the endocrine function of cultured HAECs can be assessed by detecting the above-mentioned factors in the supernatant. We found that the release of NO was not changed in the HAECs treated with 0.02 mg/ml DMSA-Fe_2_O_3_ for 24 h (Figure 3). NO released toward the vascular lumen is the most important stimulator for vascular dilator and a potent inhibitor of platelet aggregation and adhesion. NO protects against the onset and later steps in atherogenesis, and thus is one of the most important protective molecules in the vasculature. Endothelial NO synthase (eNOS) is the predominant NOS isoform in the vasculature responsible for most of the vascular NO production. A functional eNOS oxidizes its substrate l-arginine to l-citrulline and NO. Our results indicate that the eNOS function in the HAECs is not affected by treatment with 0.02 mg/ml DMSA-Fe_2_O_3_ for 24 h.

In contrast to the release of NO, the release of another vasodilator PGI-2 and the vasoconstrictor ET-1 was significantly decreased in the HAECs treated with 0.02 mg/ml DMSA-Fe_2_O_3_ for 24 h (Figure 3, *p* < 0.01 vs. control group). Besides its function as an effective vasodilator, PGI-2 can prevent platelet plug formation by inhibiting platelet activation. PGI-2 is produced in endothelial cells from prostaglandin H_2_ by the action of the enzyme PGI-2 synthase. ET-1 is secreted constitutively by endothelial cells from its inactive intermediate, big ET-1, through the action of endothelin-converting enzyme, which is present at the EC surface and on intracellular vesicles. Expression and release of PGI-2 and ET-1 in the ECs are regulated by complex signals; we did not study the mechanism for their reducing expressions and/or release in this study. However, our results demonstrate that the endocrine functions of HAECs are sensitive to DMSA-Fe_2_O_3_ treatment, and these functions may be interfered before severe cell injuries occur.

In addition to the cellular-releasing function of these vessel tone regulators, we also studied the cellular uptake function by examining the urea transporter function. The transporter for urea is expressed in the vascular endothelium that transports urea into the cell. Urea plays a significant role in the endothelial cell, and previous studies have revealed that uremic levels of urea (25 mM) inhibit l-arginine transport in cultured endothelial cells [37]. In this study, we found that the urea concentration in the HAECs treated with 0.02 mg/ml of DMSA-Fe_2_O_3_ for 24 h was significantly higher than that in control cells (Figure 3, *p* < 0.05). This observation suggests that the function of urea transporter in the HAECs is also inhibited by the DMSA-Fe_2_O_3_ exposure.

### Gene expression on HAECs

**Figure 4:**
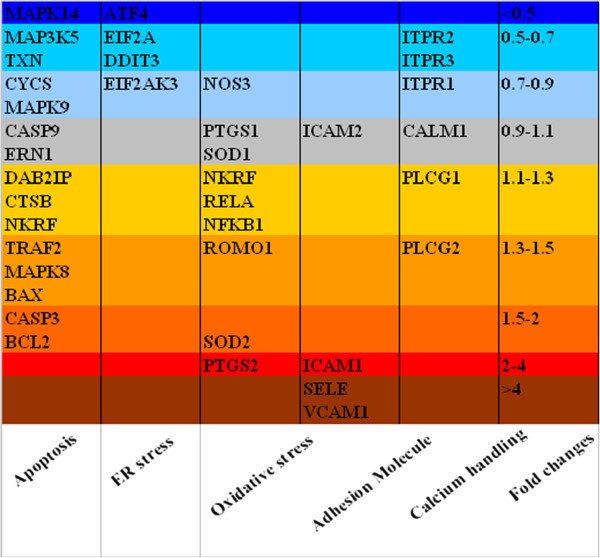
**Fold changes in gene expression: apoptosis, adhesion molecules, ER stress, oxidative stress, and calcium-handling proteins. **The changes of HAECs incubated with 0.02 mg/ml DMSA-Fe_2_O_3 _for 24 h to control the cells (HAECs without DMSA-Fe_2_O_3_) were analyzed by the 2^-ΔΔCT^ method. Gene symbols and corresponding encoded proteins: *MAP3K5*, apoptosis signal-regulating kinase 1 (ASK1); *TRAF2*, tumor necrosis factor receptor-associated factor 2 (TRAF2); *DAB2IP*, ASK1-interacting protein (AIP1); *MAPK8*, mitogen-activated protein kinase 8 (JNK1); *MAPK9*, mitogen-activated protein kinase 9 (JNK2); *MAPK14*, mitogen-activated protein kinase 14 (p38 MAPK α); *ERN1*, endoplasmic reticulum to nucleus signaling 1 (IRE1); *BCL2*, B-cell lymphoma 2 (Bcl-2); *BAX*, Bcl-2-associated X protein (Bax); *NKRF*, nuclear factor-κB repressing factor; *TXN*, thioredoxin; *CTSB*, cathespin B; *CYCS*, cytochrome C; *CASP9*, caspase-9; *CASP3*, caspase-3; *EIF2AK3*, eukaryotic translation initiation factor 2α kinase 3 (PERK); *ATF4*, activating transcription factor 4; *DDIT3*, DNA-damage-inducible transcript 3 (CHOP); *EIF2A*, eukaryotic translation initiation factor 2α; *NOS3*, nitric oxide synthase 3 (eNOS); *SOD1*, super oxide dismutase 1 (SOD-1); *SOD2*, super oxide dismutase 2 (SOD-2); *ROMO1*, reactive oxygen species modulator 1; *PTGS1*, cyclooxygenase 1 (COX-1); *PTGS2*, cyclooxygenase 2 (COX-2); *VCAM1*, vascular cell adhesion molecule 1 (VCAM-1); *ICAM1*, intercellular adhesion molecule 1(ICAM-1); *ICAM2*, intercellular adhesion molecule 2 (ICAM-2); *SELE*, endothelial-leukocyte adhesion molecule 1 (E-selectin); *PLCG1*, phospholipase C γ1; *PLCG2*, phospholipase C γ2; *ITPR1*, inositol 1,4,5-trisphosphate receptor type 1; *ITPR2*, inositol 1,4,5-trisphosphate receptor type 2; *ITPR3*, inositol 1,4,5-trisphosphate receptor type 3; *CALM1*, calmodulin 1.

Endothelial cell death, which can be caused by environmental stresses such as oxidative stress, endoplasmic reticulum stress, and adhesion molecules, is mostly apoptotic [26]. We thereby examined gene expression related to the apoptosis cascade, endoplasmic reticulum stress, oxidative stress, adhesion molecules, and calcium-handling proteins (Figure 4). After the HAECs were incubated with 0.02 mg/ml of DMSA-Fe_2_O_3_ for 24 h, the expressions of most of genes involved in the apoptosis cascade and calcium-handling proteins were changed from 0.5- to 1.5-fold compared to those of HAECs without DMSA-Fe_2_O_3_ treatment, except *MAPK14* (mitogen-activated protein kinase 14, MAPK14, also called p38-α), *CASP3* (caspase 3), and *BCL2* (Bcl-2). Caspase 3 [38] and Bcl-2 [27], which promote cell death and inhibit cell death, respectively, were increased by over 1.5-fold in mRNA expression in the experiment group. In contrast, the expression of proapoptotic *MAPK14*[39] in DMSA-Fe_2_O_3_-treated HAECs was decreased to less than 0.5-fold to that of the control cells. Therefore, the DMSA-Fe_2_O_3_ caused differential effects on the expression of pro- and anti-apoptosis genes of HAECs; this may explain why the viability of HAECs was not changed at this low concentration of DMSA-Fe_2_O_3_, which might not be sufficient to activate the cell apoptosis pathway.

In this study, the expressions of all four tested genes involved in ER stress, were down-regulated in DMSA-Fe_2_O_3_-treated HAECs (Figure 4), especially the *AFT4* gene (activating transcription factor 4), whose expression was decreased by over 50%. In contrast, most of the examined genes related to oxidative stress showed an increased change in DMSA-Fe_2_O_3_-treated HAECs with the expression of *SOD2* (superoxide dismutase 2) and *PTGS2* (cyclooxygenase-2, COX-2) elevated to 1.96- and 2.44-fold, respectively. COX-2 is unexpressed under the normal conditions but elevated during an inflammation. The data suggest that oxidative stress, not ER stress, is sensitive to DMSA-Fe_2_O_3_. In addition, the expression of *NOS3* (eNOS) was mildly decreased in DMSA-Fe_2_O_3_-treated HAECs, which was consistent to the result of NO concentration (Figure 3).

We found up-regulation of gene expression for cell-cell contact and adhesion including *ICAM1* (intercellular adhesion molecule 1, ICAM-1), *VCAM1* (vascular cell adhesion protein 1, VCAM-1), and *SELE* (endothelial-leukocyte adhesion molecule 1, E-selectin) (3.3-, 4.9-, and 8.1-fold, respectively, Figure 4). ICAM-1 is a type of intercellular adhesion molecule which continuously presents in low concentrations in the membranes of leukocytes and endothelial cells, and greatly increases upon cytokine stimulation. VCAM-1 and E-selectin are cell adhesion molecules expressed only after the endothelial cells being stimulated by cytokines and thus play an important role in inflammation. Thus, together with the data from genes associated with oxidative stress, the results of adhesion molecular genes indicate that inflammation response is likely evoked in HAECs following 0.02 mg/ml DMSA-Fe_2_O_3_ treatment before the onset of cell death.

### Effects of DMSA-Fe_2_O_3_ on HAECs tube formation

**Figure 5:**
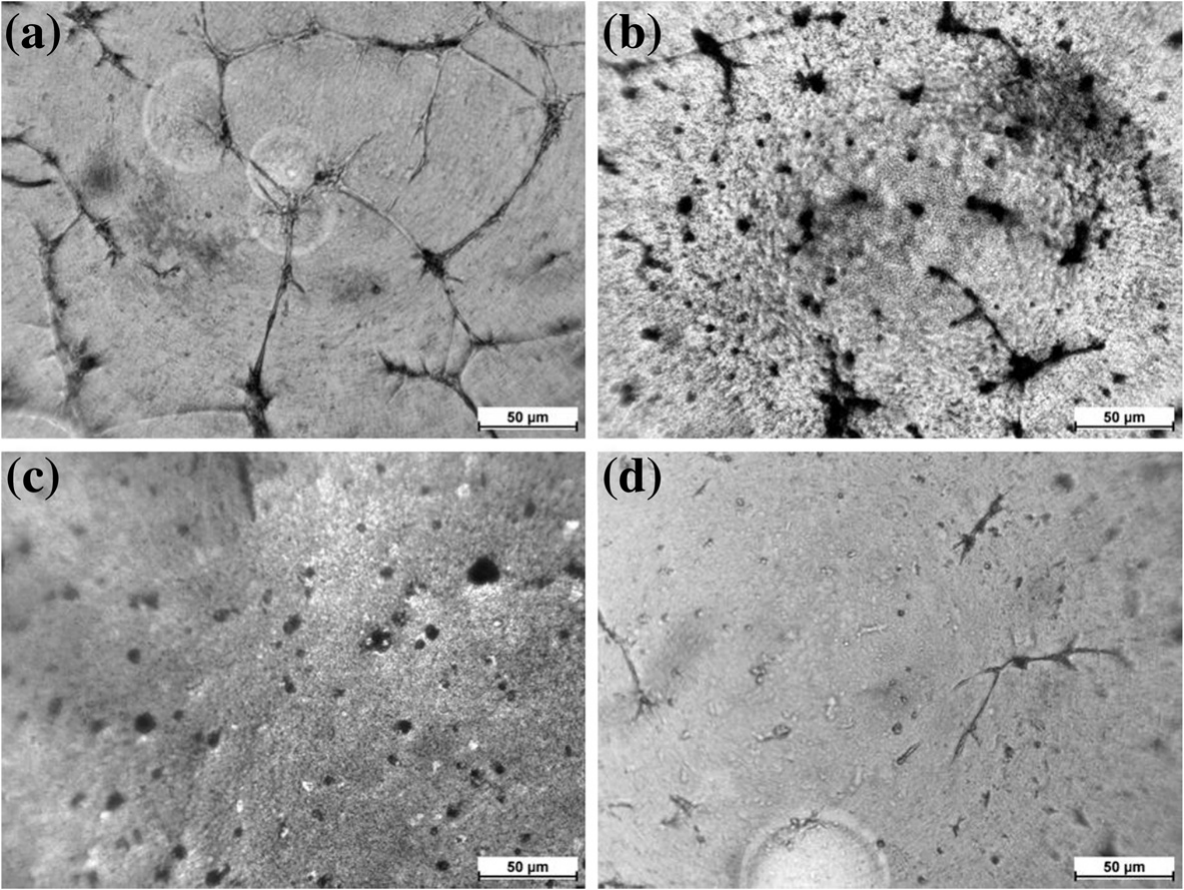
**Effect of DMSA-Fe**_**2**_**O**_**3 **_**on tube network formed by HAECs cultured on Matrigel within 14 h. **(**a**) HAECs can form a capillary-like network on Matrigel-coated wells within 14 h. (**b**) An obvious failure to form networks by HAECs in the presence of 0.01 mg/ml DMSA-Fe_2_O_3_. (**c**) Few tube networks by HAECs in the presence of 0.02 mg/ml DMSA-Fe_2_O_3_. (**d**) The high urea solution (6M urea) was used as a positive control for the inhibition of tube formation.

**Figure 6:**
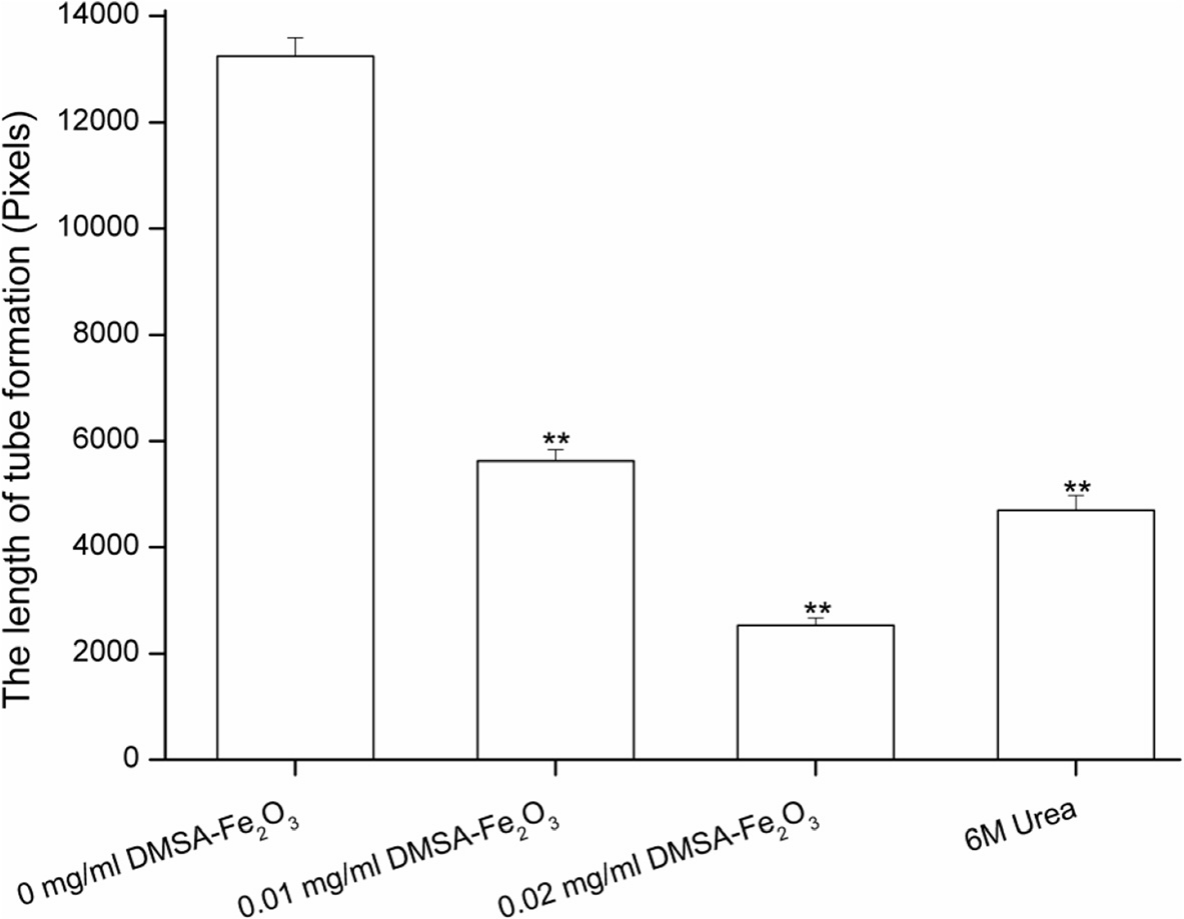
**Length of tube networks formed by HAEC cultured on Matrigel. **Image-Pro plus 6.0 for Windows software was used to measure the length of tube networks (pixels). The stained cells were inspected under a light microscope at ×100 magnification and captured more than three pictures from different fields. The average data from the same well was calculated as its quantitative value. Data are expressed as mean ± SD. **p < 0.01 vs. control.

Angiogenesis, the formation of new capillaries from preexisting blood vessels, is a motile process involving ECs activation. The migration of ECs is essential to angiogenesis and this complex process may be induced by kinds of mediators including cytokines, growth factors, and cell adhesion molecules. In physiological conditions, angiogenesis occurs in development and wound healing. However, pathological angiogenesis plays an essential role in cancer cell growth. The inhibition or antagonism of angiogenesis has been the focus of extensive basic and clinical research [40,41]. To further determine the effect of DMSA-Fe_2_O_3_ on angiogenesis by the HAECs, we performed endothelial tube formation assay using the Matrigel basement membrane matrix. We found that while HAECs without DMSA-Fe_2_O_3_ treatment formed a capillary-like network on Matrigel-coated wells within 14 h (Figure 5a), on the opposite, HAECs treated with 6M urea failed to form tubes due to its high osmolality (Figure 5d). Importantly, an obvious failure to form networks by the HAECs in the presence of DMSA-Fe_2_O_3_ with 0.01 (Figure 5b) and 0.02 mg/ml (Figure 5c) concentrations was observed. The length of the formed tube was decreased to 42.5% and 19.1% of the normal control at 0.01 and 0.02 mg/ml DMSA-Fe_2_O_3_, respectively (Figure 6). The elevated expressions of cell adhesion molecules might be responsible for the failed tube formation. The angiogenesis assay suggests that even a small amount of DMSA-Fe_2_O_3_ is also harmful to the angiogenesis of normal endothelial cells, which is an essential process in embryo development and wound healing.

## Conclusions

In summary, the present study shows that DMSA-Fe_2_O_3_ nanoparticles absorbed by the HAECs can cause a dose-dependent cytotoxic event. HAECs exposed to even a small amount of DMSA-Fe_2_O_3_ may have impaired endocrine function and angiogenic functions without obvious cell toxicity. Furthermore, the genes related to oxidative stress and inflammation response were activated. Therefore, cautious evaluation of DMSA-Fe_2_O_3_ nanoparticles *in vivo* is needed before applying them in medicine.

## Competing interests

The authors declare that they have no competing interests.

## Authors’ contributions

The work presented here was carried out in collaboration between all authors. GG, XC, and DY conceived and designed the study. GG, HW, ZB, and JX carried out the laboratory experiments. FX, YZ, and NG prepared the nanoparticles. ZG and CG co-discussed the analyses, interpretation, and presentation. GG, HW, and XC analyzed the data and interpreted the results. GG, XC, and DY wrote the paper. All authors read and approved the final manuscript.
